# The Change of a Changer: A Single Case Study of the Indigenization of a Chinese Counseling Psychologist

**DOI:** 10.3389/fpsyg.2021.625768

**Published:** 2021-10-06

**Authors:** Dongmei Wang, Baoyu Wang

**Affiliations:** ^1^Department of Psychology, School of Social and Behavioral Sciences, Nanjing University, Nanjing, China; ^2^School of Social and Behavioral Sciences, New College of Interdisciplinary Arts and Sciences, Arizona State University, Glendale, CA, United States

**Keywords:** psychotherapy, indigenization, China, change process, case study

## Abstract

Over the past several decades, the increasing popularization of psychological counseling has underlined a strong need for an indigenous approach to counseling. The current study adopted a single-case study method to construct a narrative of the indigenization process of psychotherapy in mainland China based on a comprehensive description of one prominent counseling psychologist’s experience over the past half-century. Through interviews and records of fieldwork involving the psychologist (as the case) in 10 months between 2016 and 2017, the current study analyzed the indigenization process from the following three aspects: knowledge production, counseling practice, and student training. The findings showed that there was an underlying tension between the psychologist’s traditional wisdom and his professional training in scientific psychology during the indigenization process. However, the findings of this study further revealed something missing from previous studies. First, the client-centered counselor did not assume “power” during counseling sessions, which differs from critical viewpoints in medical anthropology. Second, the students being trained underwent fundamental changes in values rather than learning a technique or resolving problems. Third, the psychologist’s life history affected his thoughts and professional practice, which occurred in a sociocultural historical context. Finally, the implications for the future direction of the indigenization of counseling practice are discussed.

## Introduction

Over the past several decades, while psychological counseling has become increasingly popular among the public in China, there has been a strong need for an indigenous approach in counseling theory and practice (Chong and Liu, 2002; Hwang, 2009; Moir-Bussy, 2012; Wu et al., 2016). Historically, since Western-style psychotherapy was first introduced in China in the early 20th century, different psychotherapy approaches have found their ways in China and played different roles in the modern history of Chinese society (Yan, 2015; Ng et al., 2017; Wang and Sang, 2019). For example, in the 1930s, psychotherapy inspired the following two schools of thought in Chinese culture: Chinese mental hygiene ideas and the indigenization of Western mental hygiene (Wang and Sang, 2019). The former concerns localizing the idea of mental hygiene, such as interpreting mental hygiene based on the theories of traditional Chinese medicine (e.g., Dong, 1928), explaining mental hygiene in terms of Buddhism (e.g., Taixu, 1938), and illustrating mental hygiene with concepts and ideas in Chinese culture (e.g., Jin, 1931; Dong, 1948a,b; Hu, 1949). Furthermore, other scholars advocated transforming traditional Chinese culture with modern mental hygiene theories. For example, Ding (1945) proposed the idea of indigenizing mental hygiene. Subsequently, some psychologists created psychotherapy theories with Chinese characteristics by incorporating ancient Chinese cultural thoughts and socialist practices in Mao’s era. Popular publications include *Cognitive Comprehension Psychotherapy* by Zhong (1988), *Comprehensive Practice Therapy* by Li (2003), *Understanding Dredging Psychotherapy* by Lu (2012), and *Taoist Cognitive Therapy* by Zhang and Yang (1998), etc.

However, these theories lost their popularity over the past several decades. On the one hand, psychotherapy theories from the previous generation are based on socialist practice, which rarely attracts consumers in the modern market economy, and on the other hand, Chinese counseling practitioners have embraced Western approaches and specialization systems. Among those who embraced Western approaches, some psychologists also attempted to enculturate Western psychotherapy by integrating it with Chinese culture (Yang, 1982, 1994, 2012; Wang, 1988, 2007, 2016; Zeng, 1997, 2002; Shen, 2001; Wong, 2002, 2011; Hsia, 2004, 2006, 2012; Moodley and West, 2005; Song, 2007, 2014; Yu et al., 2010; Shen and Gao, 2018). There are also crosscultural psychologists, such as Paul Wong, who propose integrating Chinese philosophy and indigenous psychology with counseling psychology (Wong, 2016, 2019, 2020).

Over the past two decades, the counseling market has shown great promise, as there is an increasing need for psychiatric services by the middle class in urban China. As a result, counseling services, training programs, and institutions have flourished in recent years. As psychology and counseling attract increasing attention from the public, several celebrity therapists have become public figures as they popularized psychological knowledge (Huang, 2015). Counseling has become such a fast-growing industry and a social phenomenon.

As a social and cultural phenomenon, counseling has rapidly drawn the attention of medical anthropologists interested in indigenous medical practices. These anthropologists, who are known for their critical perspective of Westernization worldwide, proposed a series of critical views of what is occurring in China in terms of the popularization of counseling practices based on Western approaches. Among these critical perspectives, the following two important concepts emerge: psychologization and individualization.

Psychologization refers to the process of reducing the political struggle against psychological processes within individuals’ minds (Parker, 2007). The term was originally used in critical psychology to describe how society frames social processes as psychological processes within individuals to legitimize the social structure (Parker, 1999, 2010; Gordo López, 2000). For example, Parker stated that “Advice about personal improvement takes the place of social transformation, and the psychologization of social life already encourages people to think that the only possible change. They could ever make would be in the way they dress and present themselves to others” (Parker, 2007, p. 2). Based on the concept of psychologization and Krieger (2009) study of how psychological education is popularized in China, Yang (2015) examined the psychologization process in China by conducting a continuous field study of psychological counseling in a factory in Changping, a district in Beijing, since 2002. Yang noted that the increase in China’s mental health problems has been inseparable from China’s comprehensive economic restructuring and wisdom since 1980. Although some people benefited from the economic reforms, many other people, especially laid-off workers, adapted to the new situation. Based on the field study, she published her book *Unknotting the Heart: Unemployment and Therapeutic Governance in China*. In this book, she noted that psychological aid in China has become a form of soft governance because it passes political–economic problems to individuals. Hizi (2018) studied a group of students in a workshop concerning interpersonal skills in Jinan. He found that the participants in the workshop could not apply what they learned in the workshop in their lives because they found that their working environments prioritized hierarchical relationships, social roles, and economic stability over individual autonomy. According to Hizi (2018), the discrepancies inside and outside of the workshop were due to market-oriented educational activities and entertainment media, which are a result of socioeconomic factors rather than psychological factors.

Huang (2014) explored how the “psycho-boom,” a term coined by medical anthropologist Kleinman (2010) to describe the emerging public need for psychological services, underlies the individualization process in China. In his view, there were three main drivers of the “psycho-boom,” namely, national policies, professional actions, and business investment. Zhang (2014; 2017; 2018) conducted a series of field studies in Yunnan investigating counseling practices. She found that psychological training and counseling practices during the “Psy fever” (a concept similar to “Psycho-boom”) played an active role in shaping the individualized self of urban middle-class Chinese. Based on his study in China, Yan (2012) associated contemporary mental health services with the individualization process he observed (e.g., emphasis on individual rights and freedom of marriage). He believed that this process indicated tension between social structures and individual agency in Chinese society.

These anthropological studies critically examined the indigenization process of psychological counseling in contemporary China. Thus, on the one hand, psycho-booms or psycho-fevers render mental health services more accessible to people; on the other hand, adopting Western approaches may create new problems, such as psychologization, or strengthen a new social phenomenon, such as individualization. These critical examinations certainly have significant implications for the future development of the counseling industry in China. However, these critical perspectives originate from anthropological researchers rather than psychologists. In addition, these studies did not cover most major approaches in counseling practices in China, such as the humanistic approach.

From a Foucauldian perspective, we might find a difference between humanistic psychology and other approaches in counseling practice, which might raise the question of whether all Western approaches lead to psychologization and individualization as previous anthropological studies found. We refer to Foucault to distinguish among different counseling approaches. In Madness and Civilization, Foucault applauded Freud as the first man to demystify madness while also criticizing how he exploited the doctor–patient structure from the previous medical model in the 19th century as follows:

“…he exploited the structure that enveloped the medical personage; he amplified its thaumaturgical virtues, preparing for its omnipotence a quasidivine status. He focused upon this single presence-concealed behind the patient and above him, in an absence that is also a total presence—all the powers that had been distributed in the collective existence of the asylum; he transformed this into an absolute observation, a pure and circumspect silence, a judge who punishes and rewards in a judgment that does not even condescend to language; he made it the mirror in which madness, in an almost motionless movement, clings to and casts off itself.” (Foucault, 1988, pp. 277–278).

While there is a stronger power relationship between the counselor and the client in psychoanalysis, notable, the power relations are different in humanistic psychology. In contrast to psychoanalysis, Carl Rogers attempted to break the “doctor–patient” structure in the therapeutic relationship. The following are the three stages of development in Roger’s counseling principle: “non-directive” therapy, “client-centered” therapy, and “person-centered” therapy; these stages demonstrate Roger’s efforts to adjust the power relationship in the counseling process (Rogers, 1989). After the end of World War II, the humanistic psychology movement, represented by person-centered therapy, Gestalt therapy, and existential psychotherapy, earned momentum in society. In the 1950s, Carl Rogers initiated the “non-directive movement,” which challenged the traditional “doctor–patient” relationship in psychotherapy (Rogers, 1951, 1957). However, compared with psychoanalysis, person-centered psychotherapy had less of an impact on psychiatry (Garfield, 1981). Nonetheless, this approach was very popular among university counseling centers (VandenBos et al., 1992).

Considering the fundamental difference in their understanding of the nature of the therapeutic relationship between humanistic psychology and other approaches (e.g., psychoanalysis), there might be a different dynamic while indigenizing humanistic psychology in China that is missing from the anthropological studies mentioned above.

In summary, the goal of the current study is to expand previous studies by: (a) reexamining previous findings from a professional psychologist’s perspective; and (b) covering the humanistic approach in China by studying a prominent counselor in the field. The existing research in anthropology provides the theoretical framework for the present study exploring the indigenization process and the relationship among the humanistic approach, psychologization and individualization.

## Research Methods

### Ethnographic Case Study

The current study was designed as an ethnographic single case study. We ground our investigation in ethnography by studying one single case, i.e., Prof. Guangrong Jiang^1^, who is a pioneer counseling psychologist.

According to Atkinson and Hammersley (1994), ethnography is characterized by (a) an emphasis on exploring the nature of social experiences instead of testing hypotheses based on *prior theories*; (b) using a variety of “unstructured” data that are not collected in a closed set of theoretical categories; (c) a comprehensive description of a small number of cases; and (d) an emphasis on the explicit interpretation of the meanings and functions of human activity. As a philosophical paradigm, Aunger (1995) argued that ethnography, as an interpretative and narrative approach, views social processes as the result of “interactions among complex intentional agents taking place in an environment of time and space.” Therefore, ethnography can describe the causal development of particular cases in detail because it places particular cases in a social, cultural, and historical context, which cannot be achieved by using formal methods (e.g., statistical methods).

In anthropology, ethnography explores the cultural meanings underlying human behaviors. The goal of psychological ethnography is to understand features of human behavior (Stewart, 2003). In our case, we attempted to understand the indigenization process a psychologist undergoes. The purpose of the study is not to generalize our results to all psychologists in China but to reveal the meaning underlying the indigenization process of a psychologist in China considering his complex social–cultural background. Differing from quantitative methods in psychology, our method is ethnographically informed, implying that the researcher collected data from participant observations, interviews, teaching records, and practice reports in a field study. The researcher interpreted the meaning of these materials to understand how indigenization occurred while considering the psychologist’s life history and personal narrative and the researcher’s own experiences, understandings, and emotional reactions (Suzuki et al., 2005).

We adopted the case study method for two reasons. First, as a methodology, case studies aim to establish the parameters of a given phenomenon (Tellis, 1997; Thomas, 2011). According to Ragin (1992).

“But what is a case? Comparative social science has a ready-made, conventionalized answer to this question: Boundaries around places and time periods define cases.”

The power of a case study is that it can provide full and thorough knowledge of the particular (Stake, 2005). Through this knowledge, we can find the parameters of the particularity, and “the parameters of the particularity are set by spatial, temporal, personal, organizational, or other factors” (Thomas, 2011). In our study, indigenization occurs in a sociocultural historical context, and our method should be able to present a rich and in-depth narrative from which we can establish the boundaries of the indigenization process by discovering the parameters (e.g., historical background, institutional factors, cultural values, etc.).

The second reason for using the case study method is that the goal of the current study is to expand the current theoretical framework to enhance our understanding of the indigenization process of Chinese counseling practice and theories. Jiang is a representative example of a counseling psychologist of his generation who was trained in Western approaches but re-encountered the thoughts of Chinese culture and started to reflect upon the necessity of the indigenization of psychological counseling theory and practice. However, as mentioned above, our reason for selecting a representative case is not to generalize our conclusions regarding the indigenization process to all psychologists in China as in an inferential statistical procedure. Instead, according to Yin (2018, “Generalizing from case studies?”, para. 2), the goal of case research is to “expand and generalize” theories and present a richer, more in-depth explanatory narrative of the subject matter based on the parameters discovered in the study (Hammersley and Gomm, 2000). Yin (2018, “Generalizing from case studies?”, para. 2) called this “analytic generalization.” In our case, the dynamic of indigenizing Western counseling approaches assumes various forms, and our goal in the selection of the case was to expand the theoretical framework established by anthropologists through a comprehensive description of a representative case (i.e., Jiang).

### Case Definition

As a single case study, we adopted the purposive sampling method; thus, we chose our case based on our research purpose (Yin, 2018). The research purpose of the current study is to examine the indigenization process from a professional psychologist’s perspective. Therefore, the criteria for the case selection were as follows: (a) he/she should be a professional psychologist in China; (b) he/she is undergoing the indigenization process as a counseling psychologist, indicating that he/she should have an educational background in the West or underwent psychology training in Western approaches); and (c) he/she has rich experience in research, practice and teaching in the field of counseling psychology in China, especially humanistic psychology. We chose Prof. Guangrong Jiang because his experience as a trained psychology researcher, practitioner, and teacher in counseling psychology fits our research purpose.

a) Jiang is recognized as a leading researcher and practitioner of humanistic psychotherapy in mainland China. Jiang’s doctoral advisor was mentored by Cecil Holden Patterson, who was a master of humanistic psychotherapy and previously worked with Carl Rogers. While he was still a doctoral student, Jiang systematically introduced Rogers’s person-centered psychotherapy in his book *The Lost and Return of Human Nature: Humanistic Psychology of Carl Rogers*. He served as the head of the clinical and counseling psychology division and the leader of the client-centered psychotherapy group under the Chinese Psychological Society. In this position, he became one of the policymakers in the field. He is a strong and active advocate for person-centered psychotherapy in China, organized a series of conferences and created 2 years of training programs in his institution.

b) Jiang earned his Ph.D. in psychology from the Chinese University of Hong Kong (CUHK) in the 1990s, when he fully embraced Western approaches (i.e., humanistic psychology) in counseling psychology. Rogers was well known for his efforts to objectively measure and analyze the therapeutic process (Melton, 1957). Jiang followed in Rogers’ footsteps by conducting systematic empirical studies to understand the psychotherapy process and outcome.

c) Jiang is a very productive researcher in psychology. According to the record in CNKI, Jiang has published 199 articles in peer-reviewed journals in China since 1987. He has mentored nearly 200 master’s and doctoral students. He founded a psychological counseling institution, as he saw that the counseling market was booming and there was a strong need for mental health services in society.

d) Jiang is working on indigenizing Rogerian principles by integrating them with Chinese culture. In recent years, he experienced a tremendous transformation during which he started to address Chinese culture in his research, teaching, and practice. This shift of focus from the Western approach to Chinese culture is very typical of his generation of psychologists who were trained in Western approaches in their early years and practiced psychology in mainland China.

e) With Jiang’s permission, the researcher received access to Jiang’s teaching, practice, and research during the 10-month field study from September 2016 to July 2017. The researcher collected data during the field study by joining Jiang’s research laboratory, working with Jiang and his students on several research projects, attending Jiang’s lectures and conference talks, participating in Jiang’s training group sessions, and taking daily field notes. Full accessibility to Jiang’s professional activities is a practical reason for choosing him as the subject of the study.

A comprehensive description and narrative explore Jiang’s practical wisdom and the relationship between his personal life and professional practice. Moreover, Jiang’s methods of researching, teaching, and practice and the interaction among these processes are narrated in depth. Through these processes, the aim is to show the dynamic change process Jiang, as a Chinese psychologist who promotes change, underwent when encountering Western culture.

### Limitations

As a research method, ethnography has the benefit of learning an insider’s perspective. Researchers are familiar with the language of the community and hence know what and how to ask to access information. However, this insider’s perspective also exposes the research to the following potential limitation: the social location and personal experience of the researcher might influence how the researcher interprets the findings (Suzuki et al., 2005). In this study, the researcher is familiar with the humanistic psychology perspective and the practice of person-centered psychotherapy. In addition, Jiang was a mentor of the researcher at a university. Therefore, the researcher is familiar with and sensitive to the humanistic perspective, which could influence how the findings were interpreted. To minimize this limitation, the researcher actively questioned the interpretations and considered alternative explanations while reviewing the findings.

Another limitation involves the theoretical framework the current study adopted to start the investigation. Our research explores the complexity of the indigenization process, but the theoretical framework in this study originated from Western intellectual traditions (e.g., modernism vs. postmodernism). The reality is that there is a lack of local theories and perspectives rooted in Chinese philosophy and society that the researcher could apply to analyze the indigenization process occurring in the field of counseling psychology.

## The Change of a 60-Year-Old: Returning to Chinese Culture

Jiang was born in the 1950s. During the cultural revolution, he experienced the freedom of “questioning everything.” He was a worker–peasant–soldier student studying music at a prestigious conservatory of music. Subsequently, he became an ideological and political cadre. In the early 1980s, influenced by the discussion of Pan Xiao, the nationwide discussion was incited by a letter called “Pan Xiao’s Letter” published in the China Youth (May 11, 1980). This is a far-reaching discussion regarding “what is the ultimate meaning of life.” This discussion was significant in modern Chinese intellectual history because it indicates that young people sought a new relationship between the self and society that differed from Mao’s ideology of sacrificing the self and serving the people (He, 2018). At that time, Jiang began to reflect upon the meaning of life. Approximately 1 year later, he furthered his studies in psychology as a graduate student exploring human nature. After graduation, he stayed in the university as a young lecturer and became one of the pioneers in the 1980s to explore the teaching and practice of psychological counseling in mainland China. In 1997, he went to Hong Kong and obtained a doctoral degree in psychological counseling.

Jiang started reading classic books because 1 day he heard a radio broadcast of the story of Wang Yangming. Wang said to his disciple “I am dying.” The disciple burst into tears upon hearing this and asked, “What are your last words, Sir?” Wang replied: “‘This mind’ is luminous and bright. What more is there to say?” Jiang was shocked and moved when he heard these words. He was about to celebrate his 60th birthday at the time. The arrival of his birthday reminded him of the passing of life. He must face the most urgent problem, i.e., death. When he started thinking about death, he heard Wang’s last words. He was astounded by the wisdom of the ancients. Hence, he found and read Wang’s complete works, and he felt “extremely humbled” after he first read the books. Why? His experiences in his early years caused him to abandon classic books. Furthermore, the reintroduction of Western science after China’s reform and opening up and his scientific training directed him to blindly follow Westerners. At this moment, he began to recognize the values of Chinese culture from personal issues. Following Wang, he began to read Confucianism, Buddhism, and Taoism. In the past 2 years, he seems to have tremendously changed. He mentioned that he enjoyed more freedom and comfort.

“Humility” occurs when people retrospectively assess their life and face challenging subjects or subjects they despised; the subjects quietly wait for them and embrace their embarrassment with an open mind and great gentleness as a mother. They were always there. In the past, people treated humility with a childlike rebellious attitude. However, people have found answers in life from these experiences when they were confused, thus resolving fatigue and anxiety in their minds and body.

Since the May Fourth Movement, the intellectual revolution and sociopolitical reform movement that occurred in China in 1917–1921, also known as the “new cultural movement,” “science” and “democracy,” have been introduced in China. With the development of modernization, the revolution of the communist ideology and politics, and the overturn of the cultural revolution, there has been a comprehensive Westernization after China’s reform and opening up. As a member of the new generation of Chinese intellectuals, Jiang followed the law of “science,” explored the truth of “science,” and examined the problems of “people” in a “scientific” way. Although Jiang (1992) published an opinion article discussing indigenizing counseling psychology in a academic journal in 1992, he did not follow up on his idea until recently. Practically, he ignored Chinese culture until he encountered a dilemma in his later years, where he found all the answers.

From being an abnegator of Chinese classics to being a humbled student of traditional wisdom, Jiang’s change of mind and his pursuit of indigenizing Western counseling approaches occurred under various circumstances. Through our analysis, we found several clues explaining this change. (a) Jiang found answers to his personal questions from Chinese intellectual wisdom rather than Western wisdom. As mentioned before, when asked about what changed his mind, he discussed his personal experience at the age of 60 when he thought about death. As melodramatic as it sounds, that was when he realized the value and power of Chinese traditional wisdom. During all these years conducting research and practicing under Western traditions, he only found satisfying answers when he returned to Chinese culture; (b) Jiang is searching for a breakthrough in psychotherapy theories. Jiang was inspired by Chinese traditional wisdom. He believes that there is much promise in integrating the wisdom of the two cultures and finding a new path for the future of psychotherapy; (c) Jiang is aware that there were problems (e.g., pathologizing) in psychotherapy in Western countries during its development. Indigenizing Western approaches also implies that we should avoid repeating these mistakes.

We explore Jiang’s psychotherapy research, practice, and teaching to discuss the complex interactions and contradictions arising among Chinese intellectuals when they encounter Western psychotherapy theory.

## Knowledge Production and Psychotherapy Practice

We delve into Jiang’s research and theories to explore how he produces knowledge of psychotherapy in his research, teaching, and practice as a Chinese psychotherapist, educator, and practitioner. Specifically, which “knowledge” should be included in psychotherapy? How does this knowledge affect practice? What are the implications?

### Research: Person or Science

According to Rogers, being a scientist and a therapist simultaneously can be puzzling. He wrote the following:

“The better therapist I have become (as I believe I have), the more I have been vaguely aware of my complete subjectivity when I am at my best in this function, And as I have become a better investigator, more “hardheaded” and more scientific (as I believe I have) I have felt an increasing discomfort at the distance between the rigorous objectivity of myself as scientist and the almost mystical subjectivity of myself as therapist.” (Rogers, 1955).

However, Jiang seems to be quite comfortable with being a scientist and a therapist simultaneously. Jiang is committed to the belief that people can be studied scientifically. He followed Rogers’ footsteps as a “hardheaded” scientist (Rogers, 1955) and conducted systematic empirical studies to understand the counseling process. Jiang published nearly 200 articles in academic journals in China and abroad since 1987. He established a reputation as a quantitative researcher in the field of counseling psychology. He used factor analysis to understand the help-seeking behavior of college students in China (Jiang and Wang, 2003); for example, he and his advisor studied students’ adaptability using hierarchical linear modeling (HLM) (Jiang and Lin, 2005).

Jiang’s commitment to quantitative inquiries of humans is highly informative regarding the following occasion. During the field study, the researcher participated in the design of a 2-year psychotherapy training program in Jiang’s newly founded counseling institution. One morning, the researcher presented a proposal to Jiang. He saw the title, “An actor in a context” and commented as follows: “We should regard people as ‘scientific.’ This proposal looks a bit like sociology.” A moment later, he added, “It’s a bit like phenomenology.” From Jiang’s reaction to the researcher’s proposal, it was apparent that Jiang distinguished between science and sociology or phenomenology. When he said, “we should regard people as scientific,” he implied that a person is an objective entity that can be studied scientifically. Since Jiang excluded sociology and phenomenology from the realm of science, it might be safe to infer that science in Jiang’s mind here mainly refers to the paradigm of positivism, which is usually characterized by quantitative approaches. To understand “science” according to Jiang, the researcher refers to the classification by Guba and Lincoln (1994).

According to Guba and Lincoln (1994), the current research paradigms of humanities and social sciences include positivism, postpositivism, critical theory, and social constructionism. These four paradigms correspond to different ontologies, epistemologies, methodologies and hence methods used in research. Therefore, the corresponding specific methods also vary. According to the above classification by Guba and Lincoln, the “science” mentioned by Jiang is a positivist model. The assumption is that “reality is objective and true,” which differs from the basic assumption of critical and constructivism theories that reality is constructed. The latter is unacceptable in Jiang’s research. The idea that a “person can be studied scientifically” indicates that a researcher should establish a hypothesis based on mathematical statistics, test the hypothesis in a large population, build mathematical models, and analyze the results. In psychology, this paradigm is also called the new Galton model (Lamiell, 2003), which is currently the dominant paradigm used to define “person” in mainstream psychology.

However, Jiang’s academic stance and attitude are also changing due to the transformation of his life influenced by traditional Chinese wisdom as mentioned above. In the following event, Jiang’s change in heart surprised his students. One day, at a regular lab meeting, the students were enthusiastically reporting their ongoing studies. To everybody’s surprise, Jiang abruptly said to the students who were discussing, “I am not very interested in what you are talking about now. I am more interested in how to use the scientific method to explain Buddhism’s ‘non-self’ theory!” Jiang’s remarks turned the meeting silent. The students were puzzled by not only Jiang’s reaction, which was very unusual, but also Jiang’s “new” ideas. Trained by Jiang in the program, no students understood why Jiang suddenly showed interest in something so different from his previous research topics. In addition, considering the academic environment and graduation requirements established by the department, the students had no intention of risking failing the program to start a project on a subject that is barely within the scope of “science.” Through later communication, Jiang mentioned that he read some Buddhist Scriptures and found the concept of “non-self” very interesting. Non-self is the core concept in Buddhism, whereas the self is the center of modern psychology. He believed that all important ideas can and should be studied in science. The results of the study might contribute to research in psychology. Nonetheless, circumstances made Jiang’s change of mind easier said than done. When the researcher found an article relevant to Jiang’s proposal and sent it to Jiang, he was excited but slightly disheartened. He replied, “Despite my great interest in the topic, I know I do not truly have the resources to do this research right now.”

One thing that made Jiang’s transformation typical is that he was still committed to the positivistic approach even when he showed interest in indigenous topics. Why did Jiang propose using positivistic methods to study indigenization and indigenous concepts even though many other researchers advocated indigenous research considering the popularity of qualitative methods in indigenous studies is not necessarily a forced choice? Through our analysis, we found that Jiang’s belief and commitment to quantitative methods were related to his personal experience of Chinese history in the 1980s.

According to Jiang, science is beyond interests, values, and even politics. In the era of the cultural revolution, psychology was once branded “pseudoscience” in China mainly for political reasons. In the late 1980s, Jiang worked as a teacher in a university. At that time, some public intellectuals left the public sphere and started a career in academia and became experts. “In particular, some young scholars who were born in the 1960s gave up their ambitions for social change, and they left for university controlled by the political system” (Xu and Luo, 2007). Similar to most intellectuals at that time, Jiang withdrew himself from the public sphere and resolved to become an expert in his field. He immersed himself in the academic world without caring about the outside world. Xu and Luo (2007) called this type of intellectuals “expert intellectuals”, referring to those intellectuals who do not engage in social movements and only focus on academic studies in their fields. Science, which is characterized by quantitative research, became an ideal place for Jiang to stay away from the public sphere.

In contrast to Rogers, Jiang did not feel that there is a conflict between the following two roles: a scientist and a therapist. In an interview, Jiang commented, “science and Tao do not conflict with each other. Tao is embedded in science whenever we do it well. If we do daily meditation in a wrong way, we depart from Tao.” Thus, Jiang believes that everything can involve the two.

### Practice: Standard Counseling Session

In his counseling practice, Jiang has a very consistent style based on the person-centered principle of the humanistic approach. The following conversation between him and a client in a session is a good example of his counseling style.

***Client:** Is my current state particularly bad?*
***Counselor:** You feel powerless and helpless. You don’t know where to go.*
***Client:** The key is that nobody knows and understands me.*
***Counselor:** You expect someone who knows you and who you can talk to. What kind of person do you wish him/her to be? Who do you want the most to become (the person)?*
***Client:** My mom? It should be my mom. But I felt that since I sought counseling, my feelings toward my mother have become complicated.*
***Counselor:** It is complicated because she is your mother?*
***Client:** She caused many of the problems, but I have a deep affection for her. I couldn’t blame her for her faults. However, I was unhappy for not blaming her, either. So, it is complicated.*
***Counselor:** Well, from an objective and rational perspective, many of your problems can be related to your mother. She raised and influenced you. And she is indeed your closest relative.*

In this conversation, the client asked the counselor, “*Is my current state particularly bad?*”, which is an interesting question. On the one hand, it is a self-reflection question. On the other hand, the client gave the counselor the right to decide. However, the counselor did not take the power and continued to motivate the client toward self-reflection as follows: “You feel powerless and helpless.” This is a typical response in Jiang’s session, which is a good example of his “let it be” counseling style. Moreover, because he believes in the person-centered principle, he focused on the client with an empathic approach, which not only affirms the client’s point of view but also transfers the power back to the client. Almost all his counseling follows this style. Nevertheless, Jiang’s care for humanity deters him from taking power in subtle moments, which is difficult to discern and easy to surpass in interpersonal relationships.

In his counseling practice, Jiang holds the person-centered principle very seriously. In the above example, it is not difficult to discern that his responses were very “empathetic,” similar to textbook statements, which exactly resembles the empathetic approach by Rogers (1951, 1957). When Carl Rogers borrowed the concept of empathy from humanities and applied it to psychotherapy in 1957, he regarded it as one of the three fundamental conditions of his proposal, which was officially marked as the core of psychotherapy. In the 1950s, Barrett-Lennard (1962) operationalized the concept at different levels of measurement and created a relationship inventory. With this scale, the empathy method could be immediately studied “scientifically.” Subsequently, Rogers’ students Robert Carkhuff, Charles Truax, and Berenson divided empathy into different levels and used these levels to train counselor professionals (Berenson and Carkhuff, 1967; Truax and Carkhuff, 1967; Carkhuff, 1969; Carkhuff and Berenson, 1977). This method has been used and is widely used in professional training and teaching in China. Jiang frequently uses this method. Because using such empathy methods in speech is unusual, novices often do not know how to respond when they are trained. They use this standard to measure their counseling performance. “Does the response sound like a counselor?” Jiang called this phenomenon the “new counselor magic chair effect.” He believes that empathetic communication involves a learning process that needs to be taken slowly. He also trained numerous professional counselors in empathy methods and was quite successful in his practical work.

In comparison to his commitment to positivist principles in research, Jiang is a staunch believer in humanistic principles in his counseling practice. The humanistic spirit that Jiang demonstrated in his practice is genuine and consistent. In addition to teaching the basic techniques, Jiang tells his students, “The critical principle of counseling is respect for humanity and in awe of life. These principles should not be lost in counseling or any other helping profession.” The researcher participated in a training session during which Jiang acted as a counselor and one of the students played the role of a client. Without a word, Jiang’s presence alone somehow had an impact on the student. The student burst into tears simply sitting in front of Jiang. During the postsession discussion, the student said that she could feel an atmosphere in which she could not avoid crying. Jiang’s strong personal presence (Brodley, 2000) in the counseling session demonstrated how deeply Jiang applied the client-centered principle in his counseling practice.

However, at the age of 60, Jiang had a change in his vision of the future of counseling psychology. Before the age of 60, Jiang believed that the future of counseling psychology lies in neuroscience and artificial intelligence. For example, he and his students developed Computerized Psychological treatments for depression (Ren and Jiang, 2014). Recently, they administered the treatment program for psychological assistance during the COVID-19 pandemic (Zhao et al., 2020). To a certain extent, these visions are based on Jiang’s commitment to positivist principles in his research practice.

However, Jiang seems to have had a change of heart in recent years. Jiang begun to emphasize the role of humanities and social sciences in the practice of counseling psychology. He once said, “The process of counseling practice is a process of qualitative research.” Thus, Jiang started to see the value of qualitative research methods in counseling psychology, which is at odds with his insistence on quantitative methods in his research practice. In addition, on different occasions, Jiang began to emphasize the search for the guiding principles of counseling practice from Chinese cultural values and traditional wisdom, which also explained Jiang’s shift in his attitudes toward qualitative research methods.

In the previous section, we mentioned that Jiang did not experience “discomfort,” and Rogers was simultaneously being an objective scientist and a subjective therapist (Rogers, 1955). Therefore, Jiang did not feel that his conviction of psychology as a hard science interferes with his humanistic counseling practice or vice versa. However, the recent change in his vision of the future of counseling psychology indicates that the underlying contradictions of the two belief systems still had an impact on Jiang’s views of the future of counseling psychology despite his subjective dismissal of the contradictions.

An understanding of the underlying distinctions between positivism and humanistic psychology in ontology and methodology might shed light on why Jiang might have had a change of heart after his transformation in recent years. First, a consensus regarding whether humanistic psychology and positivism are compatible with each other in ontology and epistemology is lacking. Nevertheless, some scholars made qualitative distinctions between the two. For example, the two views differ in their beliefs regarding whether research should (can) be value-free, how to conceptualize human nature, and the role of interpretation in research (Crotty, 1998; Waterman, 2013; DeRobertis, 2021). Considering these fundamental differences, it is not surprising that Jiang started to integrate qualitative research methods in his research practice and view a different future of counseling psychology after he found the values of traditional Chinese wisdom in his 1960s.

### Training

In addition to research and counseling practice, Jiang also includes training a new generation of Chinese professional counselors as his mission. In 2015, Jiang founded the Hubei Oriental Insight Mental Health Institution (OI), which is a private non-profit organization. By his own account, he founded this organization for three reasons. First, Jiang noticed that the psychology counseling market is booming and that there is an urgent need for professional training in counseling. Second, Jiang felt constrained by the program design at the university and sought to find a place to train practitioners in his way. Third, he sought to leave something for future generations due to his anxiety regarding death. In addition to OI, he also cooperated with other psychologists to enhance the professionalization of clinical and counseling practice in mainland China. Jiang believed that the existing graduate programs insufficiently met the needs of mental health services in China. In 2016, Jiang, along with Mingyi Qian and Fumin Fan, drafted the “Wuhan Declaration” as the initiators (see Lin et al., 2016). In the declaration, they proposed five suggestions to which improve professional training in the existing postgraduate programs in clinical and counseling psychology in China.

As a leading figure in the field, Jiang devoted himself to promoting professionalization by influencing policy-making and founding a training institution. Regarding his role as a trainer, there are two aspects to understand Jiang’s training practice, i.e., his commitment to counseling psychology and his efforts to integrate Chinese philosophy with humanistic philosophy.

On the one hand, even though OI was founded to train psychologists in all approaches, Jiang committed the training to the fundamental principles of humanistic psychology. He argues that humanism is what Chinese society currently needs. During a training session of humanistic psychotherapy, he said to his students, “the social background of China cries for humanism in particular.” In his opinion, current mainland China is similar to the West in the 1950s. “Many people seek meaning and ask themselves: ‘should such a life continue?”’ He saw the significance of humanism and humanistic psychology in a society with his diagnosis of social problems in contemporary China. “One of the most critical issues in Chinese society in the past few decades is the worship of materialism. This materialistic ethos can corrupt individuals. People have their nature. When people deviate from what is recognized by society as normal and good, they drift away from their nature.” In comparison, “humanism is unconditional” and highlights the subjectivity of human beings. Therefore, people who study humanistic theory hold a notion of “being yourself.” Thus, Jiang believes that humanism and humanistic psychotherapy can help people return to when they start to lose the sense of who they are and their nature, which is a direct consequence of materialistic cultural ethos.

Mr. Z received training in the humanistic approach in Jiang’s program at OI. He is a good example of how much impact the training has on a student in the program.

Mr. Z was a surgeon for 20 years and subsequently became a psychiatrist. In 2016, he began to study psychotherapy and participated in Jiang’s 2-year humanistic psychotherapy training program. He is the only person in the psychiatric department who underwent the training and the only doctor in the training program. He started the training seriously, speaking actively in discussion groups that promoted growth. Each time, he provided his own clinical experience based on his expertise as a “doctor.” He gradually became an “expert” in the group. The other members sought to “consult” him regarding issues, such as like mental illness symptoms and mental health laws. He was happy to provide advice and expertise. As the interaction continued, everyone began to discuss their personal experiences. People’s levels of trust and openness increased accordingly. After approximately 1 year, he revealed his loneliness, powerlessness, and depression to the group. Everyone provided support and positive feedback. He confessed that he rarely revealed these emotions, even at home. Subsequently, he gradually changed and began to be aware of his “expert” position in the group. He offered feedback to other members rather than serving as an “expert.” Immediately before the start of the fourth phase of training, he discussed his transition, which was remarkable. He revealed his understanding of the training as follows: “Everyone is unique in the world. If I express my authentic needs, I can finally speak with sincerity and consistency.” Therefore, he does not blindly follow utilitarianism, which causes him pain. However, humanism encourages everyone to make choices based on his judgment. Mr. Z acknowledged that training in humanism changed him considerably. He could express emotions that he was unable to correctly express before, which in not only improved his interpersonal relationships but also influenced how he educated his children and how he interacted with his wife. Currently, he finds that he has no hesitation to refuse to do things that violate his conscience regardless of how much interest it produces. He can express himself and do what he is willing to do. In our interview, when asked about his vision for his future, he said “I am willing to live authentically like this throughout my life.”

However, as much as he committed to the humanistic approach in his training, Jiang exerted explicit efforts to integrate humanistic philosophy with Chinese culture. The name OI (“Oriental Insight”) is an obvious example. The goal of OI is to “improve the mental health and happiness of individuals, groups, and the whole society based on oriental culture and thoughts.” Jiang has his view on how this integration could work based on his understanding of Chinese classical thoughts and humanistic philosophy. For example, he once published an article in which he argued that the Rogerian perspective of human nature is compatible with that of the Confucian view (i.e., a man at birth is fundamentally good in nature) (Jiang, 2001). Additionally, to his understanding, humanism, which placed human agency at the center of understanding humanity, is the opposite of theocentrism (God-based philosophy). Thus, as the West needs humanism, contemporary China needs humanism in modern times, which is why he believes that it is necessary to integrate humanism with traditional Chinese wisdom. In these two examples, Jiang attempted to lay a theoretical foundation for integrating humanism with traditional Chinese wisdom. However, although Jiang discussed his view of and compared humanistic philosophy and Chinese philosophy, he has not yet established a systematic theory of humanistic philosophy or Chinese philosophy. In his article concerning the compatibility of person-centered theory with Chinese culture, he mostly focused on how it is possible to apply a humanistic view in psychotherapy in counseling practice in China from a pragmatic perspective (Jiang, 2001).

Regardless of whether humanism or humanistic psychology is compatible with Chinese philosophy, Jiang attempted to convey humanistic views in local terms. Regarding the content of his teaching and training, Jiang used many examples from Chinese culture to explain theories and concepts. For example, he would explain “empathy” with the example of “Monkey King stealthily entering Princess Iron Fan’s belly,” which is a chapter in a classical novel (i.e., *Journey to the West*). Based on his teaching mode, psychotherapy theories were indigenized by Jiang before they were passed on to students. Then, the students digested the concepts through their own experiences. After constant transformation, the theories can be useful to the students. In Jiang’s pedagogy, the training focuses on not only the counseling technique but also the core ideas and values of counseling theories. Overall, these core values and ideas are the most helpful to students as demonstrated in Mr. Z’s case.

## Between Western Psychology and Chinese Culture

Our analysis of the materials from the field study showed that Jiang made an effort to integrate the Western humanistic approach with Chinese cultural values in his counseling practice, research and teaching. To understand his identity and approach, Jiang’s life and work are analyzed here.

On the one hand, Jiang is a staunch believer in human will and clients’ capacity to find meanings through their self-exploration. This faith originated from a revelation after a period of spiritual struggle in his life. Jiang underwent a spiritual crisis before he committed himself to the philosophy of humanism. When he was young, Jiang devoted himself wholeheartedly to an ideology based on the revolutionary narrative popularized by the authorities. However, as the ethos of Chinese society remarkably changed in the 1980s, revolutionary rhetoric started to lose its purchase in society. Jiang’s struggle in searching for new meanings represents a sense of disillusion and confusion shared by a generation of intellectuals. In one interview, he said,

“Our generation of people had a sincere struggled on this issue without any falsehood. I had such an unforgettable experience at the time. I still remember clearly that afternoon: after dinner, I was sitting on the lakeside. I vomited while thinking because I just could not find an answer”.

That was when Jiang found his way in humanistic psychology and then decided to attend graduate school to study counseling at The CUHK. While observing his classes and interviewing him, Jiang repeatedly told his students and the researcher that he continues to commit to the basic tenets of humanistic philosophy that: (a) psychotherapy concerns human emancipation and the goal is to help clients find their authentic selves and eventually become who they are, and (b) trust the client and rely on the power from within the client. These values helped him find meanings in life in the new era after the 1980s. Currently, he is determined to help individuals using what he learned from the West. He has been known for his empirical research in counseling psychology, such as the study of process-outcome in psychotherapy, psychological autopsy study of the suicide of Chinese college students, Chinese mental health literacy, etc.

On the other hand, Jiang’s practice is characterized by negotiation between Western and Chinese cultural values. He is more pragmatic than dogmatic in regard to providing his clients with the best service. Therefore, he frees himself from the conventional technique in humanistic therapy by adapting to clients’ needs based on cultural values in China. He is a strong advocate for indigenizing humanistic psychology. In his view, cultural theories such as “Guanxi” (relationship), “Mianzi” (face), and harmony create a hierarchical relationship between the client and the counselor, which is expected by most Chinese clients (Gao, 1996; Duan et al., 2015). Many counselors struggle with using directives in their practice as they conflict with their humanistic training (Duan et al., 2012, 2020). In a presentation, he stated the following:

“…both the counselor and the client breathe the same cultural air…. It is impossible to be a humanistic fundamentalist and, it is not necessary to pursue fundamentalism, either. We shall adopt reformism and indigenize person-centered theory. The core issue is what we should insist on and what to change.”

In practice, Jiang actively practices this conviction. For example, Jiang finds that using the humanistic approach in counseling in his practice mostly has the following two polarized prognoses: (1) the clients adopt the basic principles of humanism, and the change in perspective leads to positive therapeutic effects, or (2) the clients reject the basic principles of humanism and leave with no progress. Jiang attributed this to the collective cultural values of China. In a speech concerning the compatibility between Chinese culture and the humanistic approach in psychology, he stated the following:

“Chinese parents have a strong sense of ownership over their children. In other words, parents perceive their children as their private property. It is very different from their counterparts in the west. Chinese parents make decisions for their children. Chinese culture is characterized by collective values, which are socially oriented. In this value system, the self is minimized, and the collective is more significant. This value system is different from the client-centered approach, which emphasizes the internal evaluation of self…… Chinese culture is more likely to cultivate personalities like those who have unrealistic expectations for themselves, or someone who is always unsatisfied with their performance as well as those whose minds are filled with morals and values and feel compelled to present themselves as saints or maintain their prude.”

Nevertheless, our analysis showed that this indigenization process might be more complicated than it appears. The indigenization of humanism requires not only researchers’ conviction and conscious efforts but also a change in institutional restraint. Sometimes, structural factors hinder indigenization efforts. For example, trained in a Ph.D. program in CUHK, Jiang familiarizes himself with Western literature. When he attempted to develop a local community mental health model, he included community as a factor in his model. Subsequently, an expert in the field reminded him that the institutions where people work have a greater impact than the community on individuals’ mental health in China. Jiang then realized that he simply followed the literature from the west to search for factors to build the model.

Another example is that Jiang, in a personal conversation, mentioned that he simply cannot spare any resources to study the doctrine of “non-self” in Buddhism because he has to continue his studies of other subjects to survive in the system. The evaluation system made it impossible to pursue creative efforts (i.e., indigenous studies), which are less likely to produce desirable results. On another occasion, Jiang told the researcher that he probably has to start his indigenous study plan after he retires when he does not have to apply for funding or publish enough peer-reviewed articles to meet the requirements of the department.

## Conclusion

The purpose of the current study was to investigate a single case, Prof. Guangrong Jiang, a prominent psychologist in China, to expand the critical viewpoints from anthropological studies. These studies noted individualization and psychologization issues in the process of indigenizing counseling practice in China. The findings of this study showed a different picture of how indigenization proceeds in research, counseling practice, and training in humanistic psychology.

First, considering the person-centered value, Jiang did not take the power during the session but gave the power back to the client and let the client decide. This occurred during Jiang’s internal process of individual counseling sessions even though the client treats Jiang as an “authority.” This type of power relationship between the client and counselor distinguishes the humanistic approach from the psychoanalysis approach. Moreover, from a historical perspective, Carl Rogers proposed a revolutionary theory that completely differed from the psychoanalysis and behaviorism approach. Considering the differences among approaches, the current study enriches the breadth of the critical analysis of the indigenous process of counseling practice in China by adding humanistic viewpoints.

Second, training in a humanistic psychology program induced a shift in fundamental values among the students. Learning humanism is related to not only learning a technique or resolving problems but also a new perspective of the world and oneself. Chinese culture is characterized by collectivism such that collective interests outweigh individuals, even though it becomes increasingly individualistic. Our finding is that training in humanism facilitates students’ individualization process to separate from the traditional social values.

Third, Jiang’s life history affected his thoughts, which further shaped his research, counseling practice, and teaching. The current study revealed the complexity of the indigenization process by showing how Jiang found his way in humanism and engaged in integrating humanism with Chinese cultural values. Specifically, on the one hand, Jiang attempted to find compatibility between the basic principles of the humanistic approach and Chinese values; on the other hand, he also found fundamental conflicts between the two systems, which is a challenge humanistic psychologists face in indigenizing humanistic psychology.

To expand the current study, the transformation that Jiang underwent is experienced by not only Chinese psychologists but also all Chinese persons searching for a way of being in the contemporary era. When Jiang heard the story of Wang Yangming and then read Wang’s work, he was very inspired. This experience changed his life course, including his academic viewpoints. He no longer blindly followed the footsteps of the Western approach. Instead, he began to reflect upon the *status quo* of psychology in China.

It might not be a coincidence that Jiang’s switch to indigenous psychology was inspired by Wang Yangming’s thoughts as Wang Yangming is regarded as a master of orthodox Confucianism. Recently, some Chinese psychologists proposed that Confucian philosophy can offer indigenous roots to the psychology of social change (Li, 2014). Wong (2014) argued that Li Zehou’s “affectivity-situation ontology” or “historical ontology” provides a potential path for the future development of Confucian psychology. Wong (2014) termed Li’s theory “Four-Sentence Teaching” and stated the following:

“Human nature is from the prior human experiences. The leading structure of human nature is affectivity. The dialectic handling between situation and human affectivity is the way of Being. Practicing the way of Being is learning.”

Confucian psychology might be a promising direction for scholars who practice indigenous psychology. This path aims to establish a method that can help decide what to absorb and what to leave behind among Western psychotherapy ideas. Through this method, Chinese psychologists can digest universal ideas from Western psychotherapy and create indigenous theories rooted in the sociocultural historical context of China. In this way, psychologists can avoid separating clients from their cultural roots and create new problems because of the dismissal of their indigenous value system.

## Data Availability Statement

The original contributions presented in the study are included in the article, further inquiries can be directed to the corresponding author.

## Ethics Statement

The studies involving human participants were reviewed and approved by IRB of FJU. The ethics committee waived the requirement of written informed consent for participation. Written informed consent to participate in this study was provided by the participants. Written informed consent was obtained from the individual for the publication of any potentially identifiable images or data included in this article.

## Author Contributions

DW developed the study design, carried out the field study, collected and analyzed the data, and drafted the manuscript. BW made valuable comments and revision of the manuscript. Both authors gave their final approval of the current version of the manuscript.

## Conflict of Interest

The authors declare that the research was conducted in the absence of any commercial or financial relationships that could be construed as a potential conflict of interest.

## Publisher’s Note

All claims expressed in this article are solely those of the authors and do not necessarily represent those of their affiliated organizations, or those of the publisher, the editors and the reviewers. Any product that may be evaluated in this article, or claim that may be made by its manufacturer, is not guaranteed or endorsed by the publisher.

## Footnotes

1 According to a reviewer’s comments, the identity of Prof. Guangrong Jiang is revealed (with consent) instead of remaining confidential.
